# Ewing Sarcoma of the Mandible in an Adult: A Rare Case With Distinctive Radiologic Features

**DOI:** 10.1002/ccr3.71372

**Published:** 2025-10-26

**Authors:** Hamid Bashiri, Maryam Mohebiniya

**Affiliations:** ^1^ Department of Oral and Maxillofacial Surgery, School of Dentistry Arak University of Medical Sciences Arak Iran; ^2^ Department of Oral and Maxillofacial Radiology, School of Dentistry Arak University of Medical Sciences Arak Iran

**Keywords:** cone‐beam computed tomography, ewing sarcoma, mandible, neoplasms

## Abstract

Ewing sarcoma (ES) is a highly aggressive malignant bone tumor that rarely affects the jaw, especially in adults. This case highlights the importance of early diagnosis and referral for timely multimodal therapy—including chemotherapy, radiotherapy, and surgical resection—which is essential for improving prognosis and achieving effective management.

## 1 Case Report

A 33‐year‐old male presented with a one‐and‐a‐half‐year history of progressive swelling on the right side of the face. Clinical examination revealed a firm, mildly tender mass in the right mandibular region, resulting in facial asymmetry.

**FIGURE 1:**
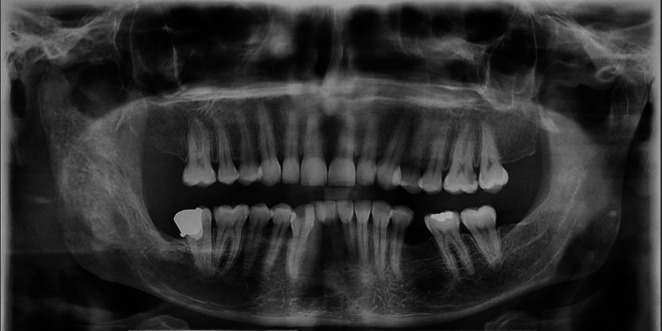
Panoramic radiograph showing an ill‐defined lesion with an altered trabecular pattern in the right mandibular ramus, suggestive of an aggressive bone lesion.

**FIGURE 2:**
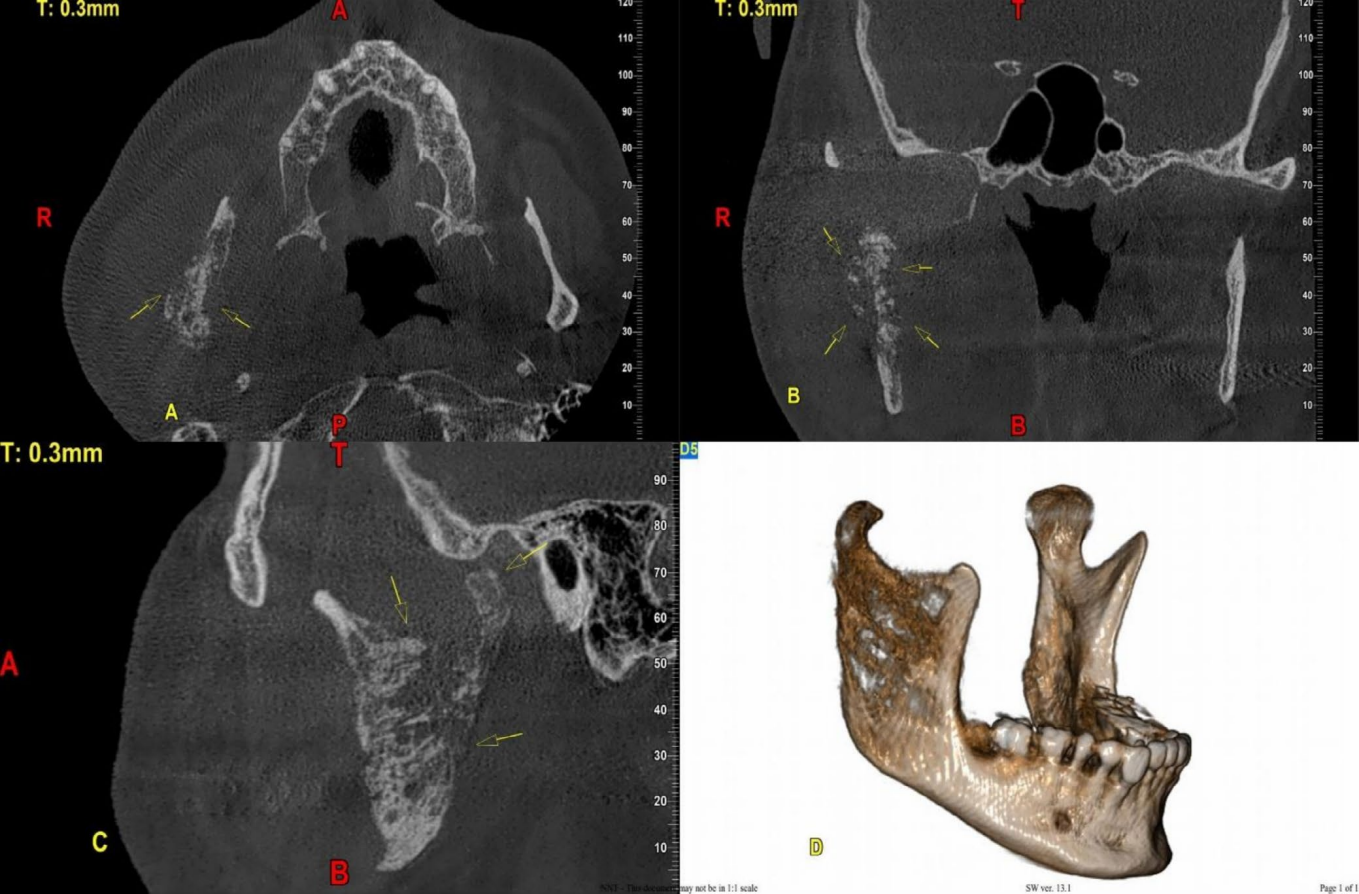
CBCT axial (A), coronal (B), sagittal (C), and 3D reconstructions (D) views show a mixed radiolucent–radiopaque expansile lesion with cortical destruction and a sunburst periosteal reaction in the right mandibular ramus.

One year prior, the patient had undergone an incisional biopsy, and histopathological and immunohistochemical (IHC) analyses confirmed the diagnosis of ES. The tumor cells were positive for CD99, CD56, and FLI1, with a Ki‐67 index of approximately 35%, consistent with a high‐grade small round blue cell tumor. Cytogenetic analysis (like EWSR1 translocation) was not performed, which represents a limitation. The diagnosis was therefore based on histopathological and IHC features, which are highly characteristic of ES. Based on this diagnosis, initial treatment with chemotherapy and radiotherapy was initiated. Despite therapy, the swelling persisted and progressively worsened, accompanied by the development of paresthesia in the affected region. This progression prompted referral to a surgeon for surgical management. Panoramic radiography and cone‐beam computed tomography (CBCT) were prescribed. Panoramic imaging demonstrated loss of normal trabecular architecture and radiographic changes in the right mandibular ramus (Figure 1). CBCT revealed a localized expansile lesion extending from the condylar and coronoid processes down to the antegonial notch vertically, and horizontally up to the distal aspect of the second molar (Figure 2).

The lesion appeared ill‐defined, with borders blending into the adjacent bone. Internally, it exhibited a heterogeneous mixed radiolucent–radiopaque appearance, characterized by a classic moth‐eaten pattern of bone destruction and areas of cortical perforation. A prominent sunburst‐type periosteal reaction was observed, indicating an aggressive osteogenic response. Three‐dimensional reconstructions confirmed marked bony expansion, surface irregularity, and soft tissue involvement, particularly in the lateral ramus, consistent with local invasion.

Ewing sarcoma (ES) is a malignant small round cell tumor, accounting for 4%–15% of primary bone tumors and ranking second in frequency after osteosarcoma among children and adolescents. It typically arises in the long bones and pelvis, with mandibular involvement being extremely rare. The radiographic features of mandibular ES may resemble other conditions. The main differential diagnoses include osteosarcoma (sunburst periosteal reaction but with osteoid matrix production), odontogenic tumors (well‐defined without periosteal reaction), and chronic osteomyelitis (moth‐eaten appearance but often linked to infection). Correlation with histopathology remains essential for definitive diagnosis [1, 2].

Definitive diagnosis requires histopathologic and IHC analysis and may be supported by cytogenetic studies [1]. In this case, a Ki‐67 index of 35% indicates high proliferative activity. ES is treated with a multimodal strategy that includes induction chemotherapy, local control (surgery and/or radiotherapy), and adjuvant chemotherapy [3]. In this case, the patient underwent chemotherapy and radiotherapy prior to surgery, which aligns with current standard protocols. Surgical resection with clear margins, followed by continued chemotherapy, is associated with improved outcomes. This approach has been shown to achieve over 70% 5‐year survival in localized disease [3].

In conclusion, although ES rarely affects the jaws, it should be considered in persistent aggressive lesions. Optimal outcomes depend on a multidisciplinary treatment approach and careful long‐term follow‐up to ensure effective management and minimize recurrence.

## Author Contributions

**Hamid Bashiri:** conceptualization, supervision, visualization, writing – review and editing. **Maryam Mohebiniya:** investigation, project administration, writing – original draft, writing – review and editing.

## Consent

Written informed consent was obtained from the patient to publish this report in accordance with the journal's patient consent policy.

## Conflicts of Interest

The authors declare no conflicts of interest.

## Data Availability

The authors have nothing to report.
